# Amygdala Structural Connectivity Is Associated With Impulsive Choice and Difficulty Quitting Smoking

**DOI:** 10.3389/fnbeh.2020.00117

**Published:** 2020-07-03

**Authors:** Ausaf A. Bari, Hiro Sparks, Simon Levinson, Bayard Wilson, Edythe D. London, Jean-Philippe Langevin, Nader Pouratian

**Affiliations:** ^1^Department of Neurosurgery, University of California, Los Angeles, Los Angeles, CA, United States; ^2^Department of Psychiatry and Biobehavioral Sciences, David Geffen School of Medicine and Semel Institute for Neuroscience and Human Behavior, University of California, Los Angeles, Los Angeles, CA, United States

**Keywords:** amygdala, probabilistic tractography, smoking cessation, human connectome, impulsivity

## Abstract

**Introduction**: The amygdala is known to play a role in mediating emotion and possibly addiction. We used probabilistic tractography (PT) to evaluate whether structural connectivity of the amygdala to the brain reward network is associated with impulsive choice and tobacco smoking.

**Methods**: Diffusion and structural MRI scans were obtained from 197 healthy subjects (45 with a history of tobacco smoking) randomly sampled from the Human Connectome database. PT was performed to assess amygdala connectivity with several brain regions. Seed masks were generated, and statistical maps of amygdala connectivity were derived. Connectivity results were correlated with a subject performance both on a delayed discounting task and whether they met specified criteria for difficulty quitting smoking.

**Results**: Amygdala connectivity was spatially segregated, with the strongest connectivity to the hippocampus, orbitofrontal cortex (OFC), and brainstem. Connectivity with the hippocampus was associated with preference for larger delayed rewards, whereas connectivity with the OFC, rostral anterior cingulate cortex (rACC), and insula were associated with preference for smaller immediate rewards. Greater nicotine dependence with difficulty quitting was associated with less hippocampal and greater brainstem connectivity. Scores on the Fagerstrom Test for Nicotine Dependence (FTND) correlated with rACC connectivity.

**Discussion**: These findings highlight the importance of the amygdala-hippocampal-ACC network in the valuation of future rewards and substance dependence. These results will help to identify potential targets for neuromodulatory therapies for addiction and related disorders.

## Introduction

The amygdala is a complex and heterogeneous structure with multiple sub-nuclei that exhibit differential connectivity with other brain regions (Swanson and Petrovich, 1998; Saygin et al., 2017) through which it mediates a wide range of behavioral responses to emotionally relevant information (Mormann et al., 2017). Although the amygdala was traditionally thought to mediate fear and aversive behavior, more recent evidence has demonstrated its role in appetitive behaviors, including reward learning, goal-directed behavior, and addiction (Wassum and Izquierdo, 2015). In rats, electrical stimulation of the basolateral nucleus of the amygdala (BLA) reinstates cocaine-seeking behavior, and inactivation of the central nucleus reduces the effect of punishment on cocaine self-administration (Xue et al., 2012). Amygdala volume also has been related to substance abuse as smaller right amygdala volumes have been associated with externalizing behaviors and cigarette smoking in adolescents (Cheetham et al., 2018).

The amygdala is a central node within a reward-related network, with connectivity to other key limbic cortical and subcortical structures that underlie reward-seeking behavior and addictive behavior. When exposed to smoking stimuli, tobacco smokers have repeatedly shown increased blood flow to a functional network involving the amygdala, nucleus accumbens (NAc), orbitofrontal cortex (OFC), hippocampus, and insula (Wilson et al., 2004; Franklin et al., 2007; Dagher et al., 2009). Imaging studies have also revealed an association between cocaine cravings and increased dopamine release in the amygdala, NAc, OFC, and anterior cingulate cortex (ACC; Koob and Volkow, 2016). Furthermore, amygdala connectivity modulates reward valuation where disconnection between the amygdala and ACC biases choices in favor of a low effort, small reward over a large reward at greater effort (Floresco and Ghods-Sharifi, 2007; Wassum and Izquierdo, 2015). Similarly, the functional disconnection between the amygdala and insula abolishes the ability to observe outcome devaluation during an instrumental conditioning task (Parkes and Balleine, 2013). Along these lines, relapse in drug addiction has been partially attributed to changes within the amygdala-hippocampus-NAc circuit. In animal models of cocaine dependence, electrical stimulation of the amygdala or the hippocampus elicits long-lasting dopamine release in the NAc which may underlie relapses in drug-seeking behavior (Blaha et al., 1997; Floresco et al., 1998; Hayes et al., 2003; Li et al., 2018). In addition to connectivity with midbrain dopaminergic neurons, amygdala connectivity to other monoaminergic nuclei within the brainstem contribute to pathological behavior. For example, the pharmacological blockade of the ventral noradrenergic bundle, which connects the amygdala to noradrenergic nuclei within the brainstem, leads to significant attenuation of heroin seeking behavior (Shaham et al., 2000; Leri et al., 2002). Connectivity of the amygdala with these structures likely influences addictive and reward-related behaviors through combined influences on reinforcement learning, reward valuation, and the subjective emotional experience associated with reward consumption.

A common feature in addiction is the preference for immediate reward even when the overall value of that reward is relatively low. This phenomenon has been formally modeled as temporal discounting in which subjects show a preference for receiving smaller immediate rewards over larger rewards in the future (McClure et al., 2004). Interpreted as a measure of impulsive choice, it has been linked with substance abuse, addiction, and relapse as well as a variety of neuropsychiatric disorders (Ahn et al., 2011; Elton et al., 2017; Owens et al., 2017). Tobacco smoking in particular has been associated with temporal discounting (Roewer et al., 2015; Ghahremani et al., 2018). Therefore, understanding the neural correlates of this behavior may yield a better understanding of the neural basis for maladaptive decision-making in individuals with various addictions, including nicotine dependence.

Previous work has shown associations between temporal discounting and several interconnected limbic structures, including the amygdala, NAc, hippocampus, OFC, parietal cortex, and ACC (Bertossi et al., 2016; Klein-Flugge et al., 2016; Frost and McNaughton, 2017; Chen et al., 2018). Here, we analyzed a large imaging and behavioral dataset to test for potential correlations between the structural connectivity of the amygdala to multiple reward-related brain regions and impulsive choice and nicotine dependence. Through this approach, we aim to evaluate the role of amygdala circuits in addictive behavior and to provide potential connectivity-based targets for future neuromodulatory therapies for nicotine dependence and other forms of addiction.

Specifically, we focus on the use of probabilistic tractography (PT) as a measure of the structural connectivity of the amygdala to other reward areas. While invasive tract tracing studies can be routinely used to study brain connectivity in animal models of addiction, their use is precluded in studies involving living human subjects. Therefore, MRI-based tractography has been used to study structural connectivity in human subjects. Increasingly, PT has been applied toward elucidating the structural organization and connectivity of the amygdala *in vivo* (Bach et al., 2011; Saygin et al., 2011), and to test for correlation of amygdala structural organization and connectivity with behavior (Greening and Mitchell, 2015; Li et al., 2018). As such, PT offers a noninvasive method of exploring how specific amygdala connections may influence addiction-related behavior and may be a promising clinical tool to evaluate potential imaging biomarkers of addiction.

## Materials and Methods

### Subjects

Data were obtained from the publicly available WU-Minn HCP 1,200 Subjects data release repository^1^ (Van Essen et al., 2013). The scanning protocol was approved by the Human Research Protection Office (HRPO), Washington University (IRB# 201 204 036). No human subject experimental procedures were undertaken at the authors’ home institution. The participants included in the HCP 1,200 Subjects data release provided written informed consent as approved by the Washington University IRB. From this repository, 200 total non-twin subjects were randomly selected. The analysis was limited to 200 subjects based on available computational resources and the costs of performing the analysis. Of these, 45 reported a history of smoking tobacco. Of the 200 total subjects, three subjects were excluded due to incomplete diffusion MRI data and without *a priori* knowledge of their smoking history. The remaining 197 subjects were included in our analyses (Table 1).

**Table 1:** Demographics of study population.

*N*	197	(%)
**Age**
22–25	41	(20.8)
26–30	91	(46.2)
31–35	64	(32.5)
36+	1	(0.5)
**Sex**
F	103	(52.3)
M	94	(47.7)

### MRI Acquisition

The data were acquired in a modified Siemens 3T Skyra scanner with a customized protocol (Sotiropoulos et al., 2013). The T1-weighted MRI has an isotropic spatial resolution of 0.7 mm, and the dMRI data have an isotropic spatial resolution of 1.25 mm. The multi-shell dMRI data were collected over 270 gradient directions distributed over three b-values (1,000, 2,000, 3,000 s/mm^2^). For each subject, the multi-shell dMRI data were collected with both L/R and R/L phase encodings using the same gradient table, which were then merged into a single copy of multi-shell dMRI data after the correction of distortions with the HCP Preprocessing Pipeline (Glasser et al., 2013).

### Probabilistic Tractography

**Figure 1:**
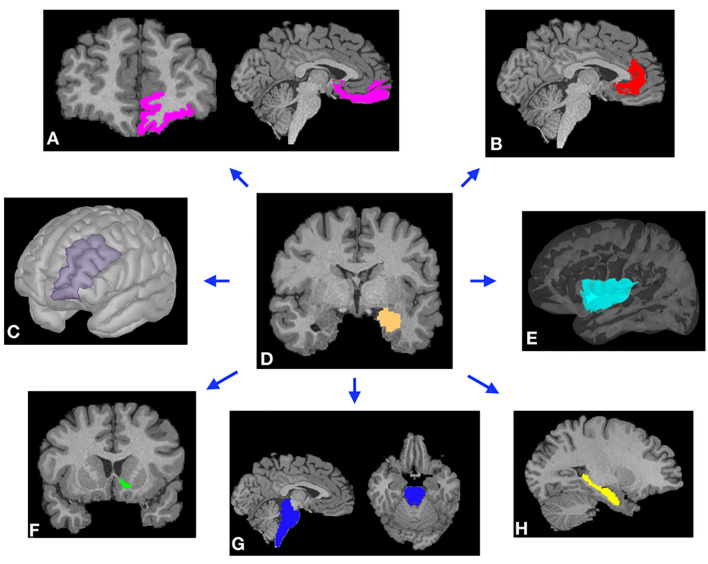
Representative examples of cortical **(A–C,E)** and subcortical **(F–H)** masks of the target brain areas and the amygdala (**D**, center) from a single subject. Masks were derived from Freesurfer automated segmentation included in the HCP dataset and were used to perform probabilistic tractography (PT) from the amygdala to each target. **(A)** Orbitofrontal cortex (OFC). **(B)** Rostral anterior cingulate cortex (rACC). **(C)** Dorsolateral prefrontal cortex (DLPFC). **(D)** Amygdala. **(E)** Insular cortex. **(F)** Nucleus accumbens (NAc). **(G)** Brainstem. **(H)** Hippocampus.

PT was performed using FSL’s FMRIB Diffusion Toolbox (probtrackx) with modified Euler streaming (Woolrich et al., 2009; Jenkinson et al., 2012). Seed and target masks were generated using the Harvard-Oxford subcortical atlas (Desikan et al., 2006). Bilateral amygdala seed mask regions of interest (ROIs) were created. Target masks included the dorsolateral prefrontal cortex (DLPFC), hippocampus, insula, NAc, OFC, rostral anterior cingulate cortex (rACC), and brainstem. The brainstem was defined as the medulla, pons, and midbrain excluding the cerebellum as depicted by the mask in Figure 1G. All tractography was performed between each (right and left) amygdala and the ipsilateral target masks except that the entire brainstem (left and right side) was used as a target for each amygdala. Each target mask was also a termination mask such that tractography was terminated once a streamline entered the target. Ipsilateral white matter masks were used as waypoints. The ventricles and cerebellum were used as exclusion masks. We used the “one way condition,” curvature 0.2, 2,000 samples, step length = 0.5, fibthresh = 0.01, distthresh = 1 and sampvox = 0.0. This resulted in 14 seed_to_target output files representing a voxelwise map of the number of seed samples from each amygdala to target. To calculate the connection probability between each amygdala voxel to each of the seven targets, we ran the FSL proj_thresh subroutine with a threshold of 1,250 on each probtrackx output. For each voxel in the seed mask with a value above the threshold, *proj_thresh* calculates the number of samples reaching each of the target masks as a proportion of the total number of samples reaching any of the target masks. This yielded a separate map of each amygdala for each target with each voxel having a value between 0 and 1 representing the connection probability of that voxel to the given target. This method normalizes connectivity within each subject, controls for expected cohort-wide variation in amygdala volume, and enables comparisons across subjects. Thus, there were 14 maps for each subject (7 targets × 2 hemispheres). To produce an overall probability of connectivity from each amygdala to target, probabilities were averaged across all voxels in each map. Next, we created a population connectivity map across all 197 subjects. Each of the previously created proj_thresh maps was registered to MNI 1 mm standard space, thresholded at a level of 0.1 and binarized. These maps were then added across all 197 subjects such that each voxel value now represented the number of subjects with connectivity to the target. FSL commands were performed using Amazon Web Services (AWS)^2^ EC2 instances running in parallel. Each AWS EC2 instance was an r4.large clone of an Amazon Machine Image (AMI) running Ubuntu 14.04 with FSL software version 5.0.10. This allowed us to run tractography on all 197 subjects simultaneously in parallel. FSL bedpostx directories for each subject and the probtrackx output files were stored on an Amazon S3 bucket.

### Behavioral Assessments

As part of the screening process, all subjects were given a comprehensive assessment of psychiatric and substance use history over the phone including the Semi-Structured Assessment for the Genetics of Alcoholism (SSAGA), which is a well-validated diagnostic instrument used in numerous previous large-scale studies, assessing a range of diagnostic categories including tobacco dependence (Kozlowski et al., 1994; Barch et al., 2013). In particular, participants were scored as either low (1 point) or high (5 points) depending on whether they met DSM criteria for tobacco dependence with difficulty quitting (“DSM tobacco dependence—difficulty quitting”). Other measures of tobacco dependence included the Fagerstrom Test for Nicotine Dependence (FTND). All tests were performed by WU-MINN HCP researchers (Barch et al., 2013). None of the test data was collected by any of the authors.

### Temporal Discounting Task

The impulsive choice was measured using a paradigm originally developed by Kirby (2009). The task identifies “indifference points” at which a person is equally likely to choose a smaller reward (e.g., $100 now) sooner rather than a larger reward ($200 in 1 year). Based on the work of Green and Myerson, an adjusting-amount approach was used in which the delay is fixed but reward amounts are adjusted on a trial-by-trial basis based on the subject’s choices (Estle et al., 2006). The area under the discounting curve (AUC_200) was used as an index of discounting (Myerson et al., 2001). All data collection was performed by the WU-MINN consortium (Barch et al., 2013).

### Statistical Analysis

All statistical analysis was carried out using the R software package^3^. One factor analysis of variance (ANOVA) was used for amygdala connectivity to target regions with Tukey HSD used for multiple comparisons correction. For analysis of the association of connectivity with impulsivity, Pearson product-moment coefficients were calculated. For testing the association between connectivity to target and difficulty quitting tobacco smoking, a two factor ANOVA model was utilized with Tukey HSD used for multiple comparisons for the interaction effect.

## Results

**Figure 2:**
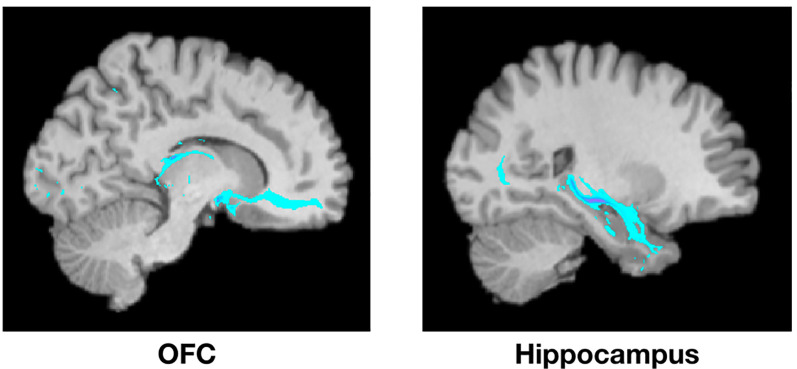
Example of PT from the amygdala to the OFC (left) and the hippocampus (right) from a single representative subject. Highlighted areas represent the number of streamlines passing through each voxel. The number of streamlines reaching each target mask was added and divided by the total number of streamlines reaching *any* of the seven target masks to calculate the probability of connectivity between the amygdala and each target.

**Figure 3:**
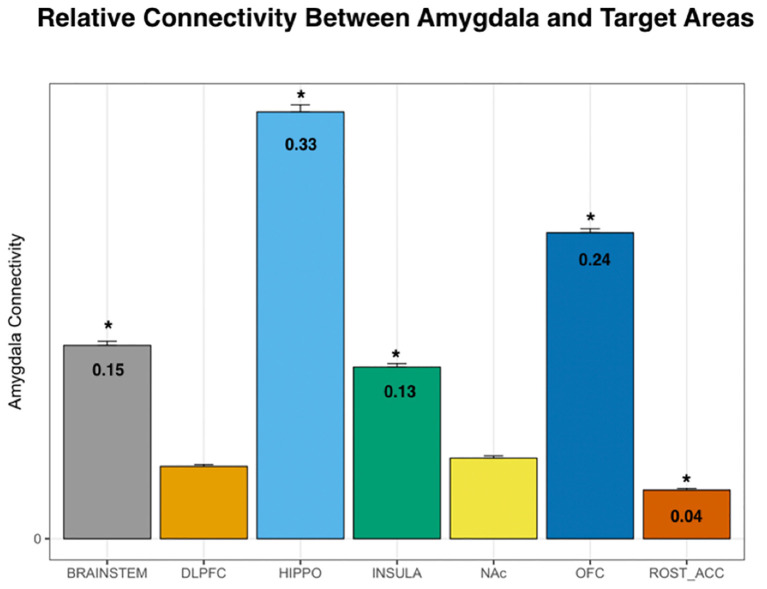
Probability of connectivity from the amygdala to each target region. Note that the highest probability of connectivity is to the hippocampus (33%). Thus, 33% of all tracks from the amygdala to the above targets terminated in the hippocampus. DLPFC, dorsolateral prefrontal cortex; HIPPO, hippocampus; NAc, nucleus accumbens; OFC, orbitofrontal cortex; ROST_ACC, rostral anterior cingulate cortex; one-way ANOVA, *p < 0.001.

Using DTI data from 197 subjects, we performed PT from the amygdala to the following seven pre-determined target structures: whole brainstem, DLPFC, hippocampus, insula, NAc, OFC, and the rACC (Figures 1, 2). The relative probability of connectivity was averaged over all amygdala voxels and this value then averaged across all subjects (Figure 3). The amygdala displayed the highest probability of connectivity with the hippocampus relative to other targets (one-way ANOVA with Tukey HSD, *n* = 2,758, *p* < 0.001 for all significant comparisons, Tables s 2, 3).

**Table 2:** ANOVA for connectivity by target.

	DF	Sum Sq.	Mean Sq.	*F*-value	Pr ( >F)
Target	6	28.21	4.702	1,267	<2e-16**
Residuals	2,751	10.21	0.004	
**p < 0.001				

**Table 3:** Tukey HSD Multiple Comparisons for mean connectivity between the amygdala and each target structure.

Target comparison	Estimate	Std. Error	*t*-value	Pr ( >|*t*|)	
DLPFC—BRAINSTEM	−0.095179	0.004341	−21.927	<0.001	**
HIPPO—BRAINSTEM	0.183838	0.004341	42.352	<0.001	**
INSULA—BRAINSTEM	−0.016964	0.004341	−3.908	= 0.00179	*
NAc—BRAINSTEM	−0.088717	0.004341	−20.439	<0.001	**
OFC—BRAINSTEM	0.088728	0.004341	20.441	<0.001	**
rACC—BRAINSTEM	−0.113867	0.004341	−26.233	<0.001	**
HIPPO—DLPFC	0.279018	0.004341	64.28	<0.001	**
INSULA—DLPFC	0.078215	0.004341	18.019	<0.001	**
NAc—DLPFC	0.006462	0.004341	1.489	= 0.75172
OFC—DLPFC	0.183907	0.004341	42.368	<0.001	**
rACC—DLPFC	−0.018688	0.004341	−4.305	<0.001	**
INSULA—HIPPO	−0.200802	0.004341	−46.261	<0.001	**
NAc—HIPPO	−0.272556	0.004341	−62.791	<0.001	**
OFC—HIPPO	−0.095111	0.004341	−21.911	<0.001	**
rACC—HIPPO	−0.297705	0.004341	−68.585	<0.001	**
NAc—INSULA	−0.071753	0.004341	−16.53	<0.001	**
OFC—INSULA	0.105692	0.004341	24.349	<0.001	**
rACC—INSULA	−0.096903	0.004341	−22.324	<0.001	**
OFC—NAc	0.177445	0.004341	40.88	<0.001	**
rACC—NAc	−0.02515	0.004341	−5.794	<0.001	**
rACC—OFC	−0.202595	0.004341	−46.674	<0.001	**
**p < 0.001, *p < 0.05					

**Figure 4:**
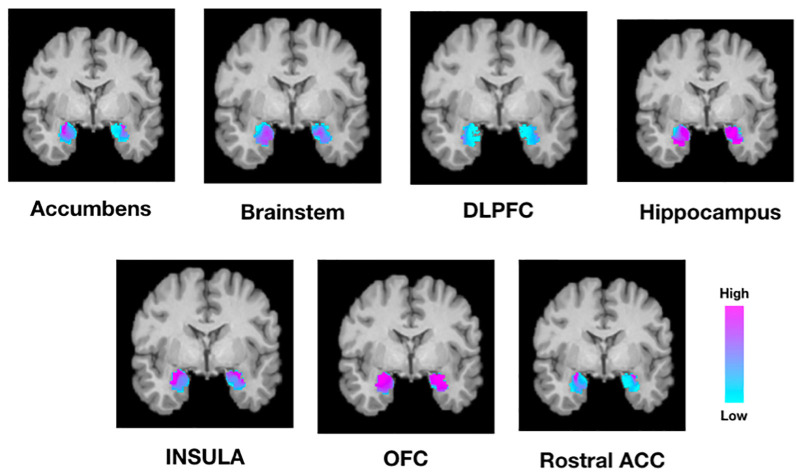
Population maps of amygdala connectivity to each target structure. The scale indicated the number of subjects that showed connectivity from each amygdala voxel to each target structure. For example, while the amygdala was homogeneously connected to the OFC and hippocampus, there was greater segregation of connectivity to the insula, rostral ACC, and nucleus accumbens. DLPFC, dorsolateral prefrontal cortex; OFC, orbitofrontal cortex; Rostral ACC, rostral anterior cingulate cortex.

Next, we tested whether connectivity of the amygdala to these target structures was spatially segregated or diffuse across all amygdala voxels. Individual connectivity maps were normalized to MNI standard space, thresholded, binarized, and summed across all subjects to determine a population connectivity map for each amygdala voxel to each target (Figure 4). We found that connectivity with the insula, NAc, DLPFC, and rACC was relatively segregated with a preference toward the dorsolateral amygdala. Meanwhile, connectivity with the brainstem and hippocampus was relatively diffuse with a localization trend toward the central, relatively medial amygdala.

**Figure 5:**
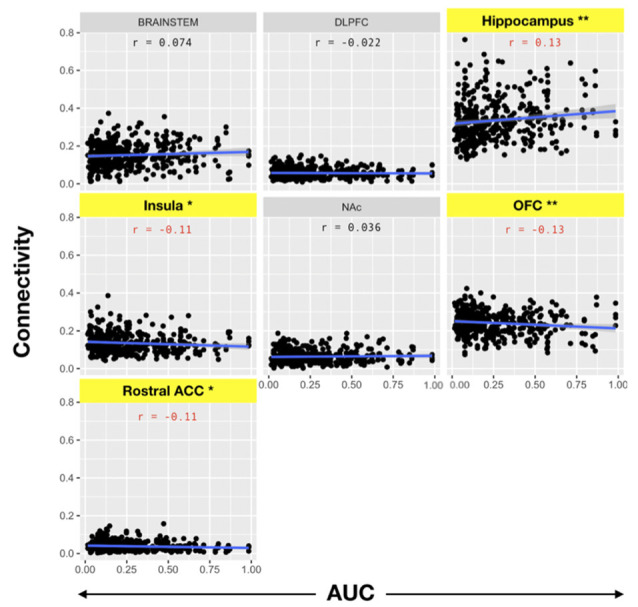
Pearson product-moment correlation of connectivity to the target structure and the area under the delay discounting curve [area under the curve (AUC)]. Connectivity between each target and the amygdala was calculated. Subsequently, the correlation coefficient between amygdala-target connectivity and the AUC of the temporal discounting behavior curve was determined. Note that AUC is inversely related to impulsive choice in that a high AUC indicates less discounting while lower AUC values indicate higher discounting or more impulsive choice. Yellow bars indicate structures with significant correlations with impulsive choice behavior. There was a significant correlation between the hippocampus (*r* = 0.13) and the AUC (i.e., decreased discounting). On the other hand, there was a significant negative correlation between connectivity with the OFC (*r* = −0.13), insula (*r* = −0.11), and rACC (*r* = −0.11) with the AUC (i.e., increased discounting; *p < 0.05, **p < 0.001).

We then evaluated the correlation between amygdala connectivity to each target structure and temporal discounting, using the area under the curve (AUC) for responses as an index of discounting reward value as a function of delay. Connectivity to the hippocampus was inversely correlated with temporal discounting whereas connectivity to the OFC, rACC, and insula were positively associated with greater preference for smaller, more immediate rewards (Pearson product-moment correlation, *n* = 197, hippocampus *r* = 0.13, *p* < 0.01; OFC *r* = −0.13, *p* < 0.01; insula *r* = −0.11, *p* < 0.05; rACC *r* = −0.11, *p* < 0.05, Figure 5, Table 4). There was no significant correlation between the AUC and connectivity with the brainstem, DLPFC, or NAc.

**Table 4:** Pearson product-moment correlation for amygdala connectivity and area under the curve (AUC) for the delay discounting task.

Target	Estimate (*r*)	*p*-value
DLPFC	−0.022	0.657
rACC	−0.106	0.036*
NAc	0.036	0.482
OFC	−0.130	0.010**
INSULA	−0.107	0.034*
HIPPO	0.130	0.010**
BRAINSTEM	0.074	0.141
**p ≤ 0.01, *p ≤ 0.05		

**Figure 6:**
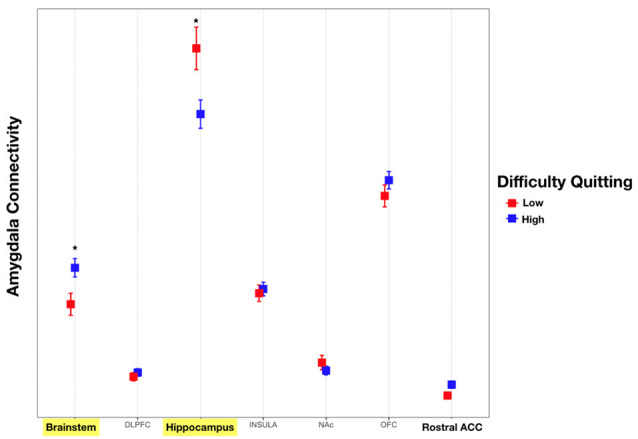
Connectivity of the amygdala with each target and difficulty quitting. The connectivity of the amygdala with each target was calculated. Subsequently, an analysis of variance (ANOVA) was calculated with connectivity as the dependent measure and target structure and difficulty quitting (low = red vs. high = blue) along with their interaction effect using Tukey HSD *post hoc* analysis. Yellow bars indicate statistically significant findings. Connectivity of the amygdala with the hippocampus was associated with less difficulty quitting while connectivity with the brainstem was associated with greater difficulty quitting (Two factor ANOVA, *n* = 45, *p < 0.01).

Addiction to nicotine, and substance abuse in general, has been previously associated with impulsivity (Moody et al., 2016; Hofmeyr et al., 2017). We sought to better characterize the role of the amygdala’s structural connectivity to other brain reward areas in mediating nicotine addiction. To this end, we utilized behavioral measures of difficulty quitting and the FTND scores as measures of severity of nicotine dependence. We found a significant interaction effect between connectivity to reward targets and tobacco dependence with difficulty quitting and FTND scores. Comparisons revealed that connectivity of the amygdala to the hippocampus was associated with low difficulty quitting and connectivity with the brainstem was associated with high difficulty quitting. There was also a trend of high difficulty quitting associated with connectivity to the OFC and rACC but this was not statistically significant [Two-factor ANOVA (Target, Level of Difficulty Quitting), *n* = 45, *p* < 0.001, Table 5, Figure 6]. Similarly, amygdala connectivity with the rACC was significantly correlated with higher FTND scores, indicative of dependence (Table 6).

**Table 5:** Tukey pairwise comparisons for an interaction effect between target and difficulty quitting smoking for both levels of the “Difficulty Quitting” factor (low vs. high).

Target	Df 1	Df 2	*F*-Ratio	*p*-value
BRAINSTEM	1	630	7.355	0.0069*
DLPFC	1	630	0.098	0.7549
HIPPO	1	630	23.879	<0.0001**
INSULA	1	630	0.097	0.7554
NAc	1	630	0.347	0.5563
OFC	1	630	1.362	0.2436
rACC	1	630	0.658	0.4177
**p < 0.001, *p < 0.01				

*Response Variable: Connectivity to Target*.

**Table 6:** Pearson product-moment correlation for amygdala-rACC connectivity and the fagerstrom test for nicotine dependence.

Target	Score	Correlation coefficient	*p*-value
rACC	FTND	0.20	0.048*
*p < 0.05			

## Discussion

In this study, we utilize PT to compare the relative structural connectivity of the amygdala to other brain areas involved in reward processing to determine the correlation between this connectivity and behaviors associated with reward processing. The results show that an amygdala-hippocampal-OFC-ACC network plays a role in the valuation of future rewards and nicotine dependence. This is one of the highest-powered studies to utilize PT to correlate the structural connectivity of the amygdala with impulsive choice and substance abuse. This technique has been used before to segment the amygdala into subnuclear components corresponding to its *in vivo* organization (Bach et al., 2011; Abivardi and Bach, 2017; Saygin et al., 2017). Our results focused on the connectivity of the amygdala to brain areas implicated in reward and decision-making, including the DLPFC, hippocampus, insular cortex, NAc, OFC, and rACC. We also included the brainstem in our analysis because of evidence that the amygdala modulates the activity of midbrain dopaminergic neurons, and receives input from brainstem nuclei, such as the locus coeruleus and nucleus of the solitary tract in mediating behavioral and autonomic responses to emotional stimuli (Veening et al., 1984; Petrov et al., 1993; Rodríguez-Ortega et al., 2017). Of the pre-selected brain targets, the amygdala had the highest probability of connectivity with the hippocampus and OFC, followed by the brainstem and insula, and lowest connectivity to the DLPFC, NAc, and rACC (Figure 3). While functional connectivity between the amygdala and all of these regions has been confirmed in prior studies, the present study is the largest to date to delineate structural connectivity to these areas and correlate connectivity with impulsive choice and measures of nicotine addiction. Furthermore, it is the first to show an association of amygdala structural connectivity with both impulsive choice and nicotine dependence. The higher probability of connectivity with the hippocampus, OFC, and brainstem are particularly interesting given that these regions have been previously implicated in smoking behavior. For example, fMRI data shows that hippocampal activation is associated with subjects assigning a higher value to future rewards (Clewett et al., 2014). A separate study identified an association between functional connectivity of the hippocampus and the ACC and a reduction in delay discounting when subjects invoked episodic future imagination (Hu et al., 2016). This association between the amygdala and memory for drug reward was corroborated by our findings of an inverse correlation between delay discounting and amygdala structural connectivity with the hippocampus (Figure 5). However, we found that connectivity with the ACC was related to preference for smaller sooner rewards. Our results support the involvement of an amygdala-hippocampal-ACC network in the valuation of future rewards. Others have proposed that the specific role of the amygdala in reward may not lie in Pavlovian or instrumental conditioned responding but rather in reward learning in the context of changing incentive values (Wassum and Izquierdo, 2015). Thus, connectivity with the ACC and hippocampus may support the structural mechanism underlying this phenomenon.

Performance on the delay discounting task has been interpreted as a measure of impulsivity and a possible model for substance abuse and relapse (Richards et al., 1999). For example, it has been shown that less temporal discounting is associated with a higher intention to quit smoking (Athamneh et al., 2017). In this article, we show that connectivity between the amygdala and hippocampus is associated with both decreased delay discounting as well as less difficulty quitting (Figures 5, 6). These findings support the concept that connectivity with the hippocampus enhances smoking cessation behavior by increasing the value of future rewards. On the other hand, connectivity with the brainstem was associated with more difficulty quitting. The brainstem is known to play a role in the neuropharmacology of nicotine and is a direct target of outputs from the central nucleus of the amygdala (Veening et al., 1984). For example, nicotine may modulate brainstem nuclei such as the ventral tegmental area, locus coeruleus, dorsal motor nucleus of the vagus, and the nucleus of the solitary tract through its activity at nicotinic receptors (Dehkordi et al., 2015). Noradrenergic signaling from the locus coeruleus to the extended amygdala is also associated with relapse to substance abuse, including smoking (Smith and Aston-Jones, 2008). Thus, structural connectivity between the amygdala and brainstem may mediate relapse to nicotine use.

In contrast to connectivity with the hippocampus, connectivity with the OFC, rACC, and insula was associated with preference for more immediate rewards (Figure 5). Based on non-human primate anatomical studies, the OFC is known to be directly connected to the amygdala (Cavada et al., 2000). The relative roles of these two structures in reward are dissociable. In rodent studies, lesions of the BLA increase preference for smaller immediate rewards, while OFC lesions paradoxically increase preference for more delayed rewards (Churchwell et al., 2009). It is known that the OFC updates the incentive value of outcomes such as the effect of time on the devaluation of future rewards (Wallis and Miller, 2003). Thus, increasing time delays result in a devaluation of the corresponding reward, with the updated value represented by the OFC (Ainslie, 1975). Here, we show that greater OFC connectivity with the amygdala is linked to a preference for more immediate rather than larger delayed rewards. This was also reflected in our finding that increased OFC connectivity was associated with more difficulty quitting smoking, although this effect did not reach statistical significance (Figure 6). We found a similar trend with connectivity to the insula and rACC with higher connectivity associated with lower AUCs reflecting a preference for more immediate choices. Also, connectivity with the rACC was associated with higher FTND scores of nicotine dependence (Table 6). Resting state fMRI has shown that functional connectivity between the insula and ACC and a monetary reward network is associated with increased discounting (Li et al., 2013). However, their analysis did not include the amygdala which may limit comparability with our data. Taken together, our structural connectivity findings support a dissociable role of the hippocampus compared to the insula/OFC/rACC in the amygdala reward network.

To further validate connectivity results and potentially inform targeting strategies for future neuromodulatory therapies, we report topographical organization patterns of amygdala connectivity (Figure 4). The amygdala is comprised of several subnuclei which have been grouped according to cytoarchitecture, neuroanatomical connectivity, and putative function. The central nucleus (CeA) and the BLA are two subnuclei that have been particularly implicated in the control of emotional processes (Cardinal et al., 2002). The BLA is constituted by the lateral (LN), basal (BN), and accessory basal nuclei (ABN) with extensive projections to the neocortex and NAc (Cardinal et al., 2002). Meanwhile, the CeA is generally thought to regulate behavioral and autonomic responses *via* strong anatomical connectivity with the brainstem (Cardinal et al., 2002). Our tractography results are broadly consistent with this organizational framework, where the NAc, DLPFC, insula, and rACC most strongly connected to lateral portions of the amygdala, while brainstem connectivity appeared relatively medial (Figure 4).

Our findings may help to inform strategies and identify potential targets of neuromodulatory therapy. Our group previously reported an association between stimulation of the BN and hedonic emotions (e.g., happiness and euphoria) in PTSD patients (Avecillas-Chasin et al., 2020). This effect may be important to note with regards to future neuromodulatory therapies for addiction, especially given previous studies describing the increase in NAc dopamine release and relapse in drug-seeking behavior with non-specific BLA stimulation (Blaha et al., 1997; Floresco et al., 1998; Hayes et al., 2003; Li et al., 2018). Taken together with the topographical and behavioral results related to amygdala connectivity, it may be preferable to target stimulation toward the LN portion of the BLA, to inhibit pathological connectivity with the insula and rACC (which were found to be associated with more impulsive decision making). Alternatively, if stimulation protocols could be designed to enhance functional connectivity, there may be benefits in targeting loci within the hippocampus to enhance beneficial communication between the hippocampus and the amygdala. Several other nodes within the tested network were found to correlate with pathological behavior and merit further investigation as potential targets of neuromodulation. For example, greater connectivity between the amygdala and the brainstem correlates with both impulsive decisions and greater difficulty quitting smoking. We previously discussed several possible brainstem nuclei which may underlie this behavioral effect. Future, studies should be directed toward identifying these nuclei and testing feasibility of targeting for neuromodulation.

The current study has several limitations. Discounting behavior and addiction are complex phenomena with multiple neurophysiological, environmental, and genetic influences. Here, we attempt to correlate complex behaviors with discrete structural imaging findings. We were limited to the HCP database which only includes limited measures of smoking dependence with the majority of subjects did not respond to this questionnaire resulting in a highly powered temporal discounting analysis, but a relatively lower powered nicotine dependence analysis. Also, the temporal discounting monetary task may have limited generalizability to substance abuse and dependence (Lopez et al., 2015), and subjects did not undergo other independent explicit impulsivity assessments such as the Barrett Impulsivity Scale 11 (BIS-11). Other prior studies have shown associations between tobacco dependence and temporal discounting (Roewer et al., 2015; Ghahremani et al., 2018). Given that our analysis was correlational, we cannot definitively make conclusions regarding causality between these correlated behavioral measures and connectivity. Finally, we are skeptical of ascribing functional significance and directionality to structural connectivity as measured by PT. Thus, while, we describe an amygdala reward network with a tendency to view connectivity as efferent projections from a central amygdala hub to our target regions, it is equally valid to view the amygdala as the target of axonal projections from these areas. Future work must integrate functional neuroimaging and invasive neurophysiological recordings to corroborate these structural connectivity findings.

## Data Availability Statement

The datasets generated for this study are available on request to the corresponding author.

## Ethics Statement

The studies involving human participants were reviewed and approved by Human Research Protection Office (HRPO), Washington University (IRB# 201 204 036). The patients/participants provided their written informed consent to participate in this study.

## Author Contributions

AB and NP were responsible for the study concept and design. HS, BW, and SL assisted with data analysis. NP, J-PL, and EL assisted with data analysis and interpretation of findings. AB drafted the manuscript. J-PL, EL, and NP provided critical revision of the manuscript for intellectual content. All authors critically reviewed content and approved the final version for publication.

## Conflict of Interest

AB Medtronic Consultant; BrainLab Consultant. NP Abbott Consultant, Fellowship Support; Medtronic Consultant, Fellowship Support; Boston Scientific Consultant; Second Sight Medical Products Consultant, Grant Support; BrainLab Grant Support.

The remaining authors declare that the research was conducted in the absence of any commercial or financial relationships that could be construed as a potential conflict of interest.

## Footnotes

^1^https://www.humanconnectome.org/

^2^http://aws.amazon.com

^3^http://www.R-project.org/
